# THE SOCIAL ROLES AND ITS RELATED FACTORS OF COMMUNITY-DWELLING OLDER ADULTS DURING THE COVID-19 PANDEMIC

**DOI:** 10.1093/geroni/igae098.2961

**Published:** 2024-12-31

**Authors:** Dai Segawa, Hayato Uchida, Ippei Suganuma, Keisuke Itotani, Atsushi Kitayama, Yuki Watanabe

**Affiliations:** Yamato University, Suita, Osaka, Japan; University of Hyogo, Himeji, Hyogo, Japan; Kyoto Tachibana University, Kyoto, Japan; Yamato University, Suita, Osaka, Japan; Iryo Sosei University, Iwaki, Fukushima, Japan; Yamato University, Suita, Osaka, Japan

## Abstract

This study focused on the social roles of community‐dwelling older adults during the COVID-19 pandemic in Japan and aimed to clarify their actual conditions and related factors. We compared the two groups of the values of four social roles of the Tokyo Metropolitan Institute of Gerontology Index of Competence and various survey items in 127 elderly subjects aged 65 years or older. The group who talked to young people tended to eat alone more (p = 0.00), to be more depressed (p = 0.00), and had lower food intake diversity scores (p = 0.04) than the group who didn’t talk to young people. No significant associations were found between the three other social roles and the values of the various survey items. The results of the Logistic regression analysis showed that the no-involvement group was significantly associated with eating alone (OR=4.28, 95%CI=1.69-10.83, p=0.00) and depression-prone individuals (OR=3.73, 95%CI=1.02-6.96, p=0.01). Tanaka et al. (2016) reported that even at the level of intergenerational interaction of “talking to younger people” in daily life, the “with opportunity” group had a 1.2 to 1.5 times lower risk of future anxiety than the “without opportunity” group. In the present study, the results support the previous studies, although the interaction was at the level of “talking to younger people. It suggested that ensuring opportunities for intergenerational exchange is important to maintain the physical and mental health of the elderly during the pandemic.

